# 6-Bromo-2-[(*E*)-thio­phen-2-yl­methyl­idene]-2,3,4,9-tetra­hydro-1*H*-carbazol-1-one

**DOI:** 10.1107/S1600536811046551

**Published:** 2011-11-12

**Authors:** R. Velmurugan, M. Sekar, A. V. Vijayasankar, P. Ramesh, M. N. Ponnuswamy

**Affiliations:** a Post Graduate and Research Department of Chemistry, Sri Ramakrishna Mission Vidyalaya College of Arts and Science, Coimbatore 641 020, India; b Department of Engineering Chemistry, Christ University, Bangalore 560 029, India; c Centre of Advanced Study in Crystallography and Biophysics, University of Madras, Guindy Campus, Chennai 600 025, India

## Abstract

In the title compound, C_17_H_12_BrNOS, the cyclo­hexene ring deviates only slightly from planarity (r.m.s. deviation for non-H atoms = 0.047 Å). In the crystal, the mol­ecules are linked into centro­symmetric *R*_2_^2^(10) dimers *via* pairs of N—H⋯O hydrogen bonds. The thio­phene ring is disordered over two positions rotated by 180° and with a site-occupation factor of 0.843 (4) for the major occupied site.

## Related literature

For the biological activity of carbazole derivatives, see: Magnus *et al.* (1992 ▶); Abraham (1975 ▶); Saxton (1983 ▶); Phillipson & Zenk (1980 ▶); Bergman & Pelcman (1990 ▶); Bonesi *et al.* (2004 ▶); Chakraborty *et al.* (1965 ▶); Kirtikar & Basu (1933 ▶); Chakraborty *et al.* (1973 ▶); Savini *et al.* (2004 ▶). For puckering parameters, see: Cremer & Pople (1975 ▶). For asymmetry parameters, see: Nardelli (1983 ▶). For hydrogen-bond motifs, see: Bernstein *et al.* (1995 ▶).
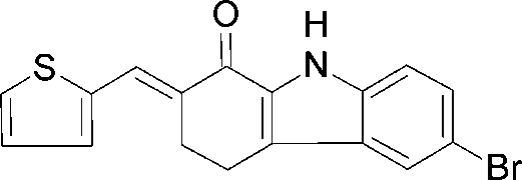


## Experimental

### 

#### Crystal data

C_17_H_12_BrNOS
*M* *_r_* = 358.25
Monoclinic, 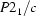

*a* = 13.8655 (5) Å
*b* = 6.3081 (3) Å
*c* = 17.4583 (7) Å
β = 103.666 (2)°
*V* = 1483.76 (11) Å^3^
*Z* = 4
Mo *K*α radiation
μ = 2.91 mm^−1^
*T* = 296 K
0.21 × 0.17 × 0.16 mm

#### Data collection

Bruker SMART APEX CCD detector diffractometer
Absorption correction: multi-scan (*SADABS*; Bruker, 1998 ▶) *T*_min_ = 0.558, *T*_max_ = 0.628
12158 measured reflections
4487 independent reflections
1953 reflections with *I* > 2σ(*I*)
*R*_int_ = 0.044

#### Refinement

*R*[*F* ^2^ > 2σ(*F* ^2^)] = 0.047
*wR*(*F* ^2^) = 0.148
*S* = 0.85
4487 reflections
191 parameters
H-atom parameters constrained
Δρ_max_ = 0.49 e Å^−3^
Δρ_min_ = −0.31 e Å^−3^

### 

Data collection: *SMART* (Bruker, 1998 ▶); cell refinement: *SAINT-Plus* (Bruker, 1998 ▶); data reduction: *SAINT-Plus*; program(s) used to solve structure: *SHELXS97* (Sheldrick, 2008 ▶); program(s) used to refine structure: *SHELXL97* (Sheldrick, 2008 ▶); molecular graphics: *ORTEP-3* (Farrugia, 1997 ▶); software used to prepare material for publication: *SHELXL97* and *PLATON* (Spek, 2009 ▶).

## Supplementary Material

Crystal structure: contains datablock(s) global, I. DOI: 10.1107/S1600536811046551/bt5684sup1.cif

Structure factors: contains datablock(s) I. DOI: 10.1107/S1600536811046551/bt5684Isup2.hkl

Supplementary material file. DOI: 10.1107/S1600536811046551/bt5684Isup3.cml

Additional supplementary materials:  crystallographic information; 3D view; checkCIF report

## Figures and Tables

**Table 1:** Hydrogen-bond geometry (Å, °)

*D*—H⋯*A*	*D*—H	H⋯*A*	*D*⋯*A*	*D*—H⋯*A*
N1—H1⋯O1^i^	0.88	2.00	2.804 (4)	151

Symmetry code: (i) 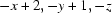.

**Fig. 1.**
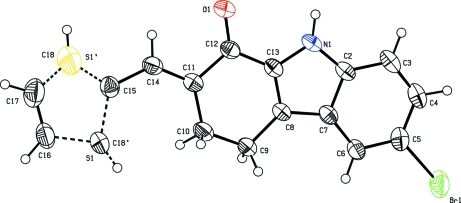
The molecular structure of the title compound, showing the atomic numbering and displacement ellipsoids drawn at the 50% probability level.

**Fig. 2.**
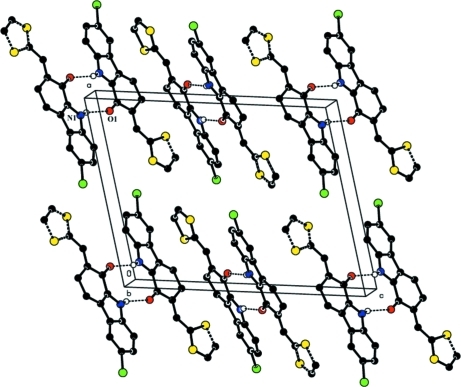
The crystal packing of the title compound. H atoms not involved in hydrogen bonding (dashed lines) have been omitted for clarity.

## supplementary crystallographic information

### Comment

Carbazole alkaloids obtained from naturally occurring sources have been the subject of extensive research, mainly because of their widespread applications in traditional medicine (Bergman & Pelcman, 1990; Bonesi *et al.*, 2004; Chakraborty *et al.*, 1965; Kirtikar & Basu, 1933). Tetrahydrocarbazole systems are present in the framework of a number of indole-type alkaloids of biological interest (Magnus *et al.*, 1992; Abraham, 1975; Saxton, 1983; Phillipson *et al.*, 1980). These types of compounds possess significant antibiotic, anti-carcinogenic, antiviral and anti-inflammatory properties (Chakraborty *et al.*, 1973). The thiophene derivatives possess the antimicrobial activity (Savini *et al.*, 2004). Against this background and to ascertain the molecular structure and conformation, the X-ray crystal structure determination of the title compound has been carried out.

The *ORTEP* plot of the molecule is shown in Fig. 1. The cyclohexene ring in the carbazole ring system adopts envelope conformation with the puckering parameters (Cremer & Pople, 1975) and the asymmetry parameters (Nardelli, 1983) are: q_2_=0.126 (5) Å, q_3_ = 0.050 (4) Å, φ_2_ = 102.0 (2)° and Δ_s_(C10 & C13)= 4.4 (5)°. Thiophene ring in the molecule is planar conformation. The sum of the bond angles around N1 [359.3°] is in accordance with *sp^2^* hybridization.

The molecules at (*x*, *y*, *z*) and (-*x* + 2, -*y* + 1, -*z*) are linked by N1—H1···O1 hydrogen bonds into a cyclic centrosymmetric *R*_2_^2^(14) dimer.

### Experimental

The mixed aldol condensation reaction of 6-bromo-1-oxo-1,2,3, 4-tetrahydrocarbazole reacted with thiophene-2-carbaldehyde in the presence of alcoholic KOH, afforded a single product, substituted 6-bromo-2- thiofuran-2-ylmethylene-2,3,4,9-tetrahydro-carbazol-1-one. This was purified by using column chromatography over silica gel (mesh 60–80). During elution of the column with petroleum ether (60–80°C) and ethyl acetate [1:2] mixture, a yellowish solid was obtained. The crystals of the title compound suitable for single XRD analysis were obtained by the slow evaporation method using the solvent mixture ethyl acetate and acetone (8:2) at room temperature.

### Refinement

N-bound H atom was located in a difference map and refined isotropically. C-bound H atoms were positioned geometrically (C–H = 0.93–0.97 Å) and allowed to ride on their parent atoms, with *U*_iso_(H) = 1.5*U*_eq_(C) for methyl H atoms and 1.2*U*_eq_(C) for all other H atoms. The thiophene ring is disordered over two positions rotated by 180 degrees and with a site occupation factor of 0.843 (4) for the major occupied site.

### Crystal data

**Table:**

C_17_H_12_BrNOS	*F*(000) = 720
*M**_r_* = 358.25	*D*_x_ = 1.604 Mg m^−^^3^
Monoclinic, *P*2_1_/*c*	Mo *K*α radiation, λ = 0.71073 Å
Hall symbol: -P 2ybc	Cell parameters from 1432 reflections
*a* = 13.8655 (5) Å	θ = 2.4–30.5°
*b* = 6.3081 (3) Å	µ = 2.91 mm^−^^1^
*c* = 17.4583 (7) Å	*T* = 296 K
β = 103.666 (2)°	Block, yellow
*V* = 1483.76 (11) Å^3^	0.21 × 0.17 × 0.16 mm
*Z* = 4	

### Data collection

**Table:**

Bruker SMART APEX CCD detector diffractometer	4487 independent reflections
Radiation source: fine-focus sealed tube	1953 reflections with *I* > 2σ(*I*)
graphite	*R*_int_ = 0.044
ω scans	θ_max_ = 30.5°, θ_min_ = 2.4°
Absorption correction: multi-scan (*SADABS*; Bruker, 1998)	*h* = −19→19
*T*_min_ = 0.558, *T*_max_ = 0.628	*k* = −4→8
12158 measured reflections	*l* = −24→24

### Refinement

**Table:**

Refinement on *F*^2^	Primary atom site location: structure-invariant direct methods
Least-squares matrix: full	Secondary atom site location: difference Fourier map
*R*[*F*^2^ > 2σ(*F*^2^)] = 0.047	Hydrogen site location: inferred from neighbouring sites
*wR*(*F*^2^) = 0.148	H-atom parameters constrained
*S* = 0.85	*w* = 1/[σ^2^(*F*_o_^2^) + (0.0752*P*)^2^ + 0.4371*P*] where *P* = (*F*_o_^2^ + 2*F*_c_^2^)/3
4487 reflections	(Δ/σ)_max_ = 0.002
191 parameters	Δρ_max_ = 0.49 e Å^−^^3^
0 restraints	Δρ_min_ = −0.31 e Å^−^^3^

### Special details

**Table:**

Geometry. All e.s.d.'s (except the e.s.d. in the dihedral angle between two l.s. planes) are estimated using the full covariance matrix. The cell e.s.d.'s are taken into account individually in the estimation of e.s.d.'s in distances, angles and torsion angles; correlations between e.s.d.'s in cell parameters are only used when they are defined by crystal symmetry. An approximate (isotropic) treatment of cell e.s.d.'s is used for estimating e.s.d.'s involving l.s. planes.
Refinement. Refinement of *F*^2^ against ALL reflections. The weighted *R*-factor *wR* and goodness of fit *S* are based on *F*^2^, conventional *R*-factors *R* are based on *F*, with *F* set to zero for negative *F*^2^. The threshold expression of *F*^2^ > σ(*F*^2^) is used only for calculating *R*-factors(gt) *etc*. and is not relevant to the choice of reflections for refinement. *R*-factors based on *F*^2^ are statistically about twice as large as those based on *F*, and *R*- factors based on ALL data will be even larger.

### Fractional atomic coordinates and isotropic or equivalent isotropic displacement parameters (Å^2^)

**Table:**

	*x*	*y*	*z*	*U*_iso_*/*U*_eq_	Occ. (<1)
Br1	1.44365 (3)	−0.32770 (7)	0.06412 (3)	0.0742 (2)	
S1	0.76193 (8)	−0.1866 (2)	0.24215 (7)	0.0665 (5)	0.843 (4)
C18'	0.76193 (8)	−0.1866 (2)	0.24215 (7)	0.0665 (5)	0.157 (4)
H18'	0.8144	−0.2822	0.2546	0.080*	0.157 (4)
O1	0.91041 (18)	0.3896 (4)	0.06209 (14)	0.0557 (6)	
N1	1.1050 (2)	0.2754 (5)	0.04571 (15)	0.0474 (7)	
H1	1.0940	0.4036	0.0258	0.057*	
C2	1.1893 (2)	0.1612 (5)	0.04737 (18)	0.0419 (8)	
C3	1.2698 (3)	0.2105 (6)	0.0147 (2)	0.0546 (10)	
H3	1.2722	0.3377	−0.0117	0.066*	
C4	1.3444 (3)	0.0657 (7)	0.0229 (2)	0.0569 (10)	
H4	1.3992	0.0949	0.0026	0.068*	
C5	1.3393 (2)	−0.1272 (6)	0.0619 (2)	0.0523 (9)	
C6	1.2619 (2)	−0.1797 (6)	0.09508 (19)	0.0458 (8)	
H6	1.2604	−0.3082	0.1209	0.055*	
C7	1.1855 (2)	−0.0306 (5)	0.08814 (16)	0.0401 (7)	
C8	1.0929 (2)	−0.0273 (5)	0.11198 (16)	0.0380 (7)	
C9	1.0491 (3)	−0.1861 (6)	0.1562 (2)	0.0556 (10)	
H9A	1.0982	−0.2242	0.2037	0.067*	
H9B	1.0334	−0.3129	0.1243	0.067*	
C10	0.9568 (3)	−0.1110 (6)	0.1789 (2)	0.0598 (10)	
H10A	0.9104	−0.2286	0.1706	0.072*	
H10B	0.9746	−0.0836	0.2352	0.072*	
C11	0.9014 (2)	0.0798 (5)	0.14016 (17)	0.0405 (7)	
C12	0.9501 (2)	0.2235 (6)	0.09254 (18)	0.0413 (8)	
C13	1.0475 (2)	0.1591 (5)	0.08458 (17)	0.0412 (8)	
C14	0.8096 (2)	0.1348 (6)	0.14535 (19)	0.0472 (8)	
H14	0.7854	0.2568	0.1174	0.057*	
C15	0.7412 (2)	0.0388 (6)	0.18652 (17)	0.0480 (9)	
C16	0.6513 (3)	−0.1697 (8)	0.2658 (2)	0.0711 (13)	
H16	0.6297	−0.2663	0.2984	0.085*	
C17	0.5980 (3)	−0.0032 (9)	0.2330 (3)	0.0820 (14)	
H17	0.5350	0.0233	0.2406	0.098*	
S1'	0.6432 (3)	0.1340 (6)	0.1851 (2)	0.0920 (14)	0.157 (4)
C18	0.6432 (3)	0.1340 (6)	0.1851 (2)	0.0920 (14)	0.843 (4)
H18	0.6164	0.2565	0.1589	0.110*	0.843 (4)

### Atomic displacement parameters (Å^2^)

**Table:**

	*U*^11^	*U*^22^	*U*^33^	*U*^12^	*U*^13^	*U*^23^
Br1	0.0607 (3)	0.0682 (3)	0.1064 (4)	−0.0012 (2)	0.0451 (2)	−0.0058 (2)
S1	0.0530 (7)	0.0737 (10)	0.0782 (8)	−0.0023 (6)	0.0263 (6)	0.0248 (7)
C18'	0.0530 (7)	0.0737 (10)	0.0782 (8)	−0.0023 (6)	0.0263 (6)	0.0248 (7)
O1	0.0592 (14)	0.0422 (14)	0.0678 (15)	0.0038 (13)	0.0193 (12)	0.0176 (13)
N1	0.0558 (17)	0.0388 (16)	0.0505 (16)	−0.0049 (14)	0.0180 (13)	0.0134 (14)
C2	0.0544 (18)	0.0353 (18)	0.0381 (16)	−0.0101 (17)	0.0152 (14)	0.0008 (15)
C3	0.064 (2)	0.053 (2)	0.0515 (19)	−0.019 (2)	0.0240 (17)	0.0035 (18)
C4	0.058 (2)	0.064 (3)	0.057 (2)	−0.017 (2)	0.0289 (17)	−0.003 (2)
C5	0.0466 (18)	0.057 (2)	0.057 (2)	−0.0071 (18)	0.0210 (16)	−0.0063 (19)
C6	0.0471 (18)	0.044 (2)	0.0508 (18)	−0.0068 (17)	0.0199 (15)	0.0014 (17)
C7	0.0489 (17)	0.0386 (19)	0.0349 (15)	−0.0072 (16)	0.0141 (14)	−0.0016 (15)
C8	0.0455 (16)	0.0348 (18)	0.0352 (15)	−0.0046 (15)	0.0127 (13)	0.0018 (15)
C9	0.054 (2)	0.049 (2)	0.071 (2)	0.0043 (18)	0.0288 (18)	0.0206 (19)
C10	0.066 (2)	0.054 (2)	0.071 (2)	0.009 (2)	0.0374 (19)	0.023 (2)
C11	0.0510 (18)	0.0354 (18)	0.0364 (15)	−0.0037 (16)	0.0129 (14)	−0.0020 (15)
C12	0.0490 (18)	0.0353 (19)	0.0395 (16)	−0.0046 (16)	0.0099 (14)	−0.0004 (16)
C13	0.0471 (17)	0.0367 (19)	0.0408 (16)	−0.0073 (16)	0.0122 (14)	0.0006 (16)
C14	0.0522 (19)	0.044 (2)	0.0455 (18)	−0.0011 (17)	0.0112 (15)	0.0072 (16)
C15	0.0454 (17)	0.059 (2)	0.0402 (16)	−0.0049 (18)	0.0108 (14)	0.0017 (17)
C16	0.052 (2)	0.093 (4)	0.073 (3)	−0.014 (2)	0.022 (2)	0.016 (3)
C17	0.056 (2)	0.117 (4)	0.079 (3)	0.004 (3)	0.027 (2)	0.005 (3)
S1'	0.084 (2)	0.106 (3)	0.093 (2)	−0.006 (2)	0.0363 (18)	0.011 (2)
C18	0.084 (2)	0.106 (3)	0.093 (2)	−0.006 (2)	0.0363 (18)	0.011 (2)

### Geometric parameters (Å, °)

**Table:**

Br1—C5	1.915 (4)	C8—C9	1.480 (4)
S1—C16	1.683 (4)	C9—C10	1.503 (4)
S1—C15	1.707 (4)	C9—H9A	0.9700
O1—C12	1.243 (4)	C9—H9B	0.9700
N1—C2	1.368 (4)	C10—C11	1.500 (5)
N1—C13	1.375 (4)	C10—H10A	0.9700
N1—H1	0.8789	C10—H10B	0.9700
C2—C3	1.403 (4)	C11—C14	1.343 (4)
C2—C7	1.411 (4)	C11—C12	1.495 (4)
C3—C4	1.361 (5)	C12—C13	1.448 (4)
C3—H3	0.9300	C14—C15	1.451 (4)
C4—C5	1.403 (5)	C14—H14	0.9300
C4—H4	0.9300	C15—S1'	1.480 (4)
C5—C6	1.376 (4)	C16—C17	1.333 (6)
C6—C7	1.400 (4)	C16—H16	0.9300
C6—H6	0.9300	C17—S1'	1.445 (6)
C7—C8	1.441 (4)	C17—H17	0.9300
C8—C13	1.366 (4)		
			
C16—S1—C15	92.6 (2)	H9A—C9—H9B	107.7
C2—N1—C13	107.5 (3)	C11—C10—C9	120.7 (3)
C2—N1—H1	124.1	C11—C10—H10A	107.1
C13—N1—H1	128.2	C9—C10—H10A	107.1
N1—C2—C3	129.2 (3)	C11—C10—H10B	107.1
N1—C2—C7	109.2 (3)	C9—C10—H10B	107.1
C3—C2—C7	121.5 (3)	H10A—C10—H10B	106.8
C4—C3—C2	117.7 (3)	C14—C11—C12	116.2 (3)
C4—C3—H3	121.1	C14—C11—C10	124.7 (3)
C2—C3—H3	121.1	C12—C11—C10	119.1 (3)
C3—C4—C5	120.6 (3)	O1—C12—C13	121.6 (3)
C3—C4—H4	119.7	O1—C12—C11	122.5 (3)
C5—C4—H4	119.7	C13—C12—C11	115.9 (3)
C6—C5—C4	123.1 (3)	C8—C13—N1	111.1 (3)
C6—C5—Br1	119.5 (3)	C8—C13—C12	124.8 (3)
C4—C5—Br1	117.3 (2)	N1—C13—C12	124.1 (3)
C5—C6—C7	116.8 (3)	C11—C14—C15	131.8 (3)
C5—C6—H6	121.6	C11—C14—H14	114.1
C7—C6—H6	121.6	C15—C14—H14	114.1
C6—C7—C2	120.1 (3)	C14—C15—S1'	121.8 (3)
C6—C7—C8	133.8 (3)	C14—C15—S1	126.0 (3)
C2—C7—C8	106.0 (3)	S1'—C15—S1	112.2 (2)
C13—C8—C7	106.3 (3)	C17—C16—S1	112.9 (3)
C13—C8—C9	123.6 (3)	C17—C16—H16	123.6
C7—C8—C9	130.1 (3)	S1—C16—H16	123.6
C8—C9—C10	113.8 (3)	C16—C17—S1'	116.6 (4)
C8—C9—H9A	108.8	C16—C17—H17	121.7
C10—C9—H9A	108.8	S1'—C17—H17	121.7
C8—C9—H9B	108.8	C17—S1'—C15	105.6 (3)
C10—C9—H9B	108.8		
			
C13—N1—C2—C3	178.8 (3)	C10—C11—C12—O1	−176.4 (3)
C13—N1—C2—C7	−0.2 (3)	C14—C11—C12—C13	−177.7 (3)
N1—C2—C3—C4	−178.3 (3)	C10—C11—C12—C13	2.9 (4)
C7—C2—C3—C4	0.6 (5)	C7—C8—C13—N1	−0.6 (3)
C2—C3—C4—C5	1.0 (5)	C9—C8—C13—N1	−180.0 (3)
C3—C4—C5—C6	−1.5 (6)	C7—C8—C13—C12	177.2 (3)
C3—C4—C5—Br1	175.9 (3)	C9—C8—C13—C12	−2.2 (5)
C4—C5—C6—C7	0.4 (5)	C2—N1—C13—C8	0.5 (4)
Br1—C5—C6—C7	−176.9 (2)	C2—N1—C13—C12	−177.4 (3)
C5—C6—C7—C2	1.1 (5)	O1—C12—C13—C8	−175.6 (3)
C5—C6—C7—C8	177.9 (3)	C11—C12—C13—C8	5.2 (4)
N1—C2—C7—C6	177.4 (3)	O1—C12—C13—N1	2.0 (5)
C3—C2—C7—C6	−1.6 (5)	C11—C12—C13—N1	−177.3 (3)
N1—C2—C7—C8	−0.2 (3)	C12—C11—C14—C15	−178.8 (3)
C3—C2—C7—C8	−179.3 (3)	C10—C11—C14—C15	0.6 (6)
C6—C7—C8—C13	−176.7 (3)	C11—C14—C15—S1'	178.2 (4)
C2—C7—C8—C13	0.5 (3)	C11—C14—C15—S1	−0.6 (6)
C6—C7—C8—C9	2.7 (6)	C16—S1—C15—C14	178.6 (3)
C2—C7—C8—C9	179.8 (3)	C16—S1—C15—S1'	−0.4 (3)
C13—C8—C9—C10	−8.5 (5)	C15—S1—C16—C17	0.8 (4)
C7—C8—C9—C10	172.2 (3)	S1—C16—C17—S1'	−1.1 (6)
C8—C9—C10—C11	16.1 (5)	C16—C17—S1'—C15	0.8 (5)
C9—C10—C11—C14	166.8 (4)	C14—C15—S1'—C17	−179.2 (3)
C9—C10—C11—C12	−13.8 (5)	S1—C15—S1'—C17	−0.2 (4)
C14—C11—C12—O1	3.0 (5)		

### Hydrogen-bond geometry (Å, °)

**Table:**

*D*—H···*A*	*D*—H	H···*A*	*D*···*A*	*D*—H···*A*
C14—H14···O1	0.93	2.33	2.759 (4)	108.
N1—H1···O1^i^	0.88	2.00	2.804 (4)	151.

Symmetry codes:  (i) −*x*+2, −*y*+1, −*z*.
